# Flow Resistance Analysis of Clinically Significant Portal Hypertension in Patients with Liver Cirrhosis

**DOI:** 10.1155/2022/9396371

**Published:** 2022-09-26

**Authors:** Yizhe Wang, Luxiang Zhao, Zhuozhao Zheng, Yu Zhang

**Affiliations:** ^1^Department of Radiology, Beijing Tsinghua Changgung Hospital, School of Medicine, Tsinghua University, Beijing 102218, China; ^2^School of Mechanical Engineering, State Key Laboratory of Tribology, Tsinghua University, Beijing 100084, China; ^3^School of Medicine, Tsinghua University, Beijing 100084, China

## Abstract

Cirrhosis-induced clinically significant portal hypertension (CSPH) is a fatal disease. Early detection of CSPH is vitally important to reduce the patients' mortality rate. In this study, combined with three-dimensional image construction technology and computational fluid dynamics (CFD), an image-based flow resistance analysis was proposed. The flow resistance analysis was performed for nine cirrhosis patients with CSPH and ten participants without liver diseases, respectively. The results showed that the flow resistance coefficient of the portal vein system in CSPH patients was significantly lower than that in the control group (0.97 ± 0.11 Pa/(mL/s) for CSPH patients; 1.80 ± 0.40 Pa/(mL/s) for the control group; *P* = 0.028). In contrast, although main portal vein dilation was found in CSPH patients, the cross-sectional area enlargement was not statistically significant (186.01 ± 57.48 mm^2^ for CSPH patients; 166.26 ± 33.74 mm^2^ for the control group; *P* = 0.39). The research outcomes indicated that the flow resistance analysis was more sensitive than the commonly used vessel size measurement in the detection of CSPH. In summary, we suggest using flow resistance analysis as a supplementary noninvasive method to detect cirrhosis patients with CSPH.

## 1. Introduction

**Figure 1:**
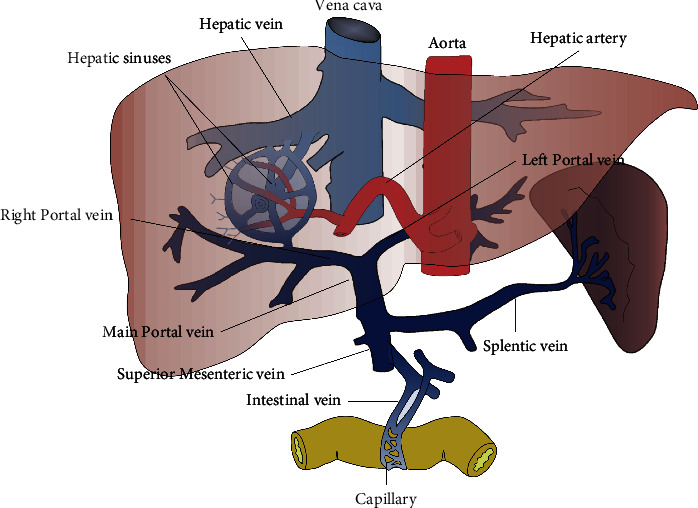
Anatomic structure of PV system.

The portal vein (PV) is a channel system through which blood flows into the liver. The PV is located between two capillary networks. One end is the capillary network of the stomach, intestine, spleen, and pancreas, and the other end is the hepatic sinusoid. The main portal vein (MPV) is formed by the joining of the superior mesenteric vein (SMV) and splenic vein (SV). MPV is further divided into a left portal vein branch (LPV) and a right portal vein branch (RPV), which enter the left and right lobes of the liver, respectively, and then successively bifurcate to form its terminal branches in the hepatic sinusoids [1]. The PV system is illustrated in Figure 1. When liver cirrhosis occurs, it causes high resistance to the blood flowing through the portal vein to the liver, resulting in a continuous increase in portal vein pressure, that is, portal hypertension.

Portal hypertension is a common clinical syndrome defined as a pathological increase in portal pressure, and more than 80% are sinus portal hypertension caused by liver cirrhosis.

For sinus portal hypertension dominated by hepatitis cirrhosis or alcoholic cirrhosis, the currently accepted gold standard is the measurement of the hepatic venous pressure gradient (HVPG), by which portal hypertension is defined as an HVPG of at least 5 mmHg. Once the HVPG increases to greater than 10 mmHg, it is defined as clinically significant portal hypertension (CSPH), easy to cause serious complications such as bleeding from esophageal and gastric varices [2, 3]. However, the measurement of HVPG is invasive and available only in a limited number of hospitals due to technical difficulties. To meet the clinical need, there has been growing interest in developing noninvasive diagnostic methods to detect CSPH [4–6].

In recent years, medical image-based computational fluid dynamics (CFD) has demonstrated significant potential in the noninvasive diagnosis of vascular diseases. However, to date, CFD simulations are primarily performed to simulate arterial diseases such as intracranial aneurysms, carotid artery stenosis, and coronary artery diseases [7–12]. In contrast to the abundance of arterial studies, studies about computational models of PV are limited [13, 14]. Applying CFD to PV is difficult because the resulting venous images are not as clear as arterial images, flow boundary conditions vary for each patient, and the comparisons with clinical evidence are insufficient to justify CFD estimations. Therefore, more PV-specific CFD technologies need to be developed.

The objective of the current study was to propose a CFD-based flow resistance analysis method for the detection of CSPH in cirrhosis patients. Flow resistance studies are advantageous because the flow resistance coefficients are free of boundary conditions in the CFD calculations and they are calculated by only relying on the three-dimensional (3D) shape of the portal vein displayed by enhanced CT and thus convenient in clinical practice.

## 2. Materials and Methods

### 2.1. Patient Information and Computed Tomography Angiography (CTA)

Nine patients with liver cirrhosis (seven males, two females; mean BMI = 23.9 kg/m^2^; ages 42-91 with a median age of 63.5) were included in this study. Their main clinical and biochemical characteristics are presented in Table 1. After overnight fasting, all the patients were subjected to a transient ultrasound test using the FibroScan® 502 Touch to acquire their liver stiffness (LS) values [15, 16]. The LS values were measured at least two times on different days, of which the average value was taken. The obtained results were expressed in kilopascals (kPa). The control group consisted of ten patients (seven males, three females; mean BMI = 25 kg/m^2^; ages 38-64 with a median age of 54.3) who had no previous or current diagnosis of liver disease.

All the participants were subjected to abdominal enhanced CT imaging, which was performed using either a Discovery CT 750 HD scanner (GE Healthcare, Waukesha, Wisconsin, USA) or a CT 760 (United Imaging, Shanghai, China). Scans were completed with the participants in the supine position. All participants received the median cubital vein administration of 90 ml contrast media (Omnipaque 350, GE Healthcare, Shanghai, China) at a rate of 3 mL/s. After the start of injection of the contrast medium, an abdominal CT scan was conducted in the arterial phase at 35 s, in the portal vein phase at 70 s, and in the delayed phase at 180 s, respectively. The following parameters were used: slice thickness, 1.25 mm; pixel size, 0.684 mm × 0.684 mm; tube voltage, 120 kVp; tube current, 150-300 mA; and matrix size, 512 × 512.

In this study, all nine patients had been confirmed with LS >20 kPa. In patients with liver cirrhosis caused by nonviral, all showed definite collateral circulation on enhanced CT imaging. Based on the Baveno VI consensus and comprehensive clinical manifestations, these cirrhosis patients were diagnosed with CSPH [17, 18].

### 2.2. Flow Resistance Coefficients (*A* and *B*)

In light of Darcy–Weisbach equation that is typically used to quantitatively describe flow resistance [19], the pressure drops caused by friction along a specified pipe length to the average velocity of fluid flow can be determined. Assuming laminar flow, pressure drops are calculated as follows:

(1) $$\begin{array}{c} \Delta p=\frac{1}{2}\lambda \left(\frac{L}{D}\right)\rho u^{2}, \end{array}$$

where Δ*p* denotes the pressure drop, *u* is the blood velocity *λ* = (64/*RE*) = (64*μ*/*ρuD*), *μ* is the viscosity of the fluid, *ρ* is the blood density, *L* is the tube length, and *D* is the tube size. In the current study, blood flow was assumed to be an incompressible fluid; hence, the blood flow velocity represents the volumetric flow rate. Substituting the blood flow velocity with the volumetric flow rate, Equation (1) can be re-expressed as follows:

(2) $$\begin{array}{c} \Delta p=32\mu \left(\frac{L}{D^{2}}\right)u=BQ, \end{array}$$

where *Q* denotes the volumetric flow rate inside the tube, and *B* denotes the linear flow resistance coefficient, which is associated only with the tube diameter and length. If the tube is not straight but exhibits abrupt morphological changes such as stenosis, narrowing, and curvature, then an additional term is required to calculate the pressure drop for those specific elements, as follows:

(3) $$\begin{array}{c} \Delta p=\frac{1}{2}\xi \rho u^{2}=AQ^{2}, \end{array}$$

where *ξ* denotes a constant for a specific shape, such as the curvature, and *A* denotes the quadratic flow resistance coefficient. In this case, the pressure drop in a generally curved tube can be expressed as follows:

(4) $$\begin{array}{c} \Delta p=AQ^{2}+BQ. \end{array}$$

For a certain section of the vein, *A* and *B* are constant values. The same volume of blood flowing through different vessels results in different pressure drops; high-pressure drops always result in high values of *A* and *B*. It is noteworthy that the values of *A* and *B* are determined merely by the vessel configuration and not by the bloodstream flowing through the vessel. Since our focus is on vessel dilation, only flow resistance coefficient *B* will be discussed.

The aforementioned flow resistance analysis has been successfully performed to estimate intracranial bypass surgeries [20]. However, it is noteworthy that Equation (4) is accurate only under the assumption that the vessels are not in pulsatile movement, which is acceptable for venous analyses because the venous pulsatile change is typically negligible during a cardiac cycle [13].

### 2.3. CTA-based CFD Simulation

The geometry of the PV was constructed using digital imaging and communications in medicine files of the portal vein phase. Contour interpolation was performed to generate two-dimensional (2D) contours from the greyscales of the pixels. A 3D geometry was constructed via the interpolation of the 2D contours in the normal direction. The conservation equations for 3D flows with rigid walls were solved using an open-source CFD solver (https://www.openfoam.org) [21] in which the diffusion term was discretized using a second-order mathematical scheme. The reliability of OpenFOAM has been validated previously in multiple CFD areas, including hemodynamic simulations [21, 22]. Blood was assumed to be a Newtonian fluid with a density of 1050 kg/m^3^ and a dynamic viscosity of 0.0036 Pa∙s [20], which is consistent with assumptions in the literature [23, 24].

Mesh independence was evaluated in previous studies involving arterial simulations, wherein the mesh scale was smaller than 0.4 mm for vessels with a diameter of a few millimeters for generating more than 1 million computational cells; this ensured that the CFD results are free of the grid number [21]. In this study, we generated a tetrahedron/hexahedron combined mesh with a mesh scale smaller than 0.2 mm, which resulted in an average mesh number of 6 million across all the cases. At near-wall regions, boundary-fitted prism layers were generated at the boundaries to improve the resolution of the relevant scales in a fluid motion. To calculate the flow resistance coefficient *B*, multiple pressure drops were imposed at the inlet and outlet of each tested vessel.

### 2.4. Flow Resistance Analysis of the PV System

**Figure 2:**
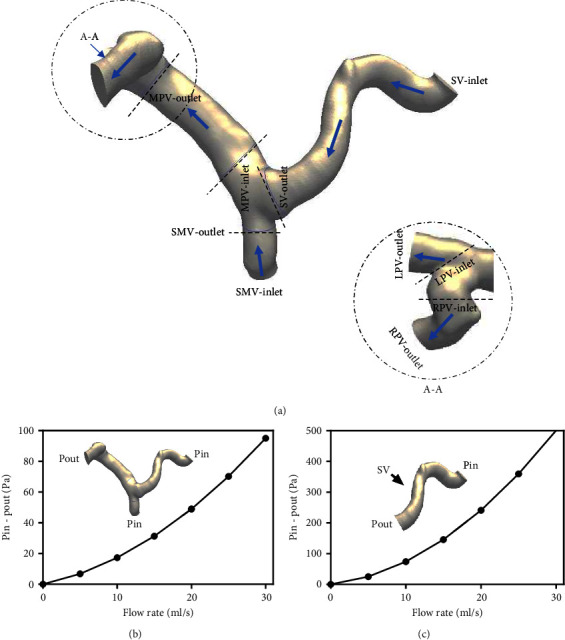
Example of portal vein segmentation and flow resistance calculation. (a) PV segmentations. (b) Overall flow resistance of PV (*A* = 0.072 Pa/(mL/s)2; *B* = 1 Pa/(mL/s)). (c) Flow resistance of SV (*A* = 0.745 Pa/(mL/s)^2^; *B* = 2.7 Pa/(mL/s)).

The entire PV system can be further separated into SV, SMV, MPV, RPV, and LPV, as shown in Figure 2(a), in which the dotted line indicates the locations of the inlet and outlet of each branch, whereas the arrows indicate the blood flow directions. In CFD simulations, when the PV is simulated as an integral vessel, the overall flow resistance is obtained, as shown in Figure 2(b). When the flow resistance of each branch is simulated separately, the flow resistance of each branch is obtained. For example, the flow resistance of the SV can be obtained from the calculation of the pressure drops under different blood flow rates at the SV, as shown in Figure 2(c). Figure 2(b) and 2(c) indicate that exact boundary conditions are not necessary to obtain accurate flow resistance coefficients *A* and *B*, since they are constant parameters for a specified section of the vessel.

### 2.5. Cross-Sectional Area Analysis of the MPV

In clinical practice, the cross-sectional area of the MPV is often used as a diagnostic marker for portal hypertension [25]. In our study, two radiologists separately measured the cross-sectional area of the MPV at the CT image postprocessing workstation. A cross-sectional area was recorded at three positions for each MPV, located at both ends of the MPV and the midpoint of the MPV, respectively, and then the averaged value was adopted.

## 3. Results

### 3.1. Flow Resistance Analysis vs. Cross-Sectional Area Analysis

**Figure 3:**
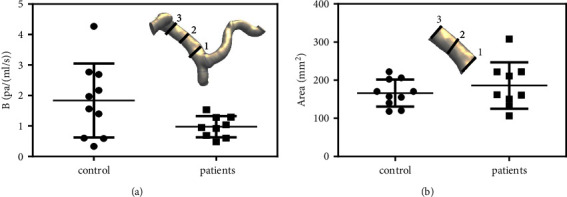
Flow resistance of PV and cross-sectional analysis of MPV. (a) Flow resistance of PV. (b) Cross-sectional analysis of MPV.

The entire PV can be regarded as an integral vessel, in which the total flow resistance reflects the overall effect of cirrhosis on the blood entering the liver. An advantage of the CFD simulation is that the total flow resistance of the entire PV can be estimated. As shown in Figure 3(a), the total flow resistance coefficient *B* of the patients (0.97 ± 0.11 Pa/(mL/s)) was lower than that of the control group (1.80 ± 0.40 Pa/(mL/s)). Further *t*-tests showed *P*=0.028, confirming statistical significance between the two groups.

The clinically applied cross-sectional area measurement is shown in Figure 3(b). The results showed that the cross-sectional areas were 186.01 ± 57.48 mm^2^ and 166.26 ± 33.74 mm^2^ for the patients and the control group, respectively. Further *t*-tests showed *P*=0.39, indicating that the enlargement of MPA was not statistically significant. Compared to the flow resistance analysis, the cross-sectional area measurement was less sensitive in identifying patients with CSPH.

### 3.2. Flow Resistance Analyses of Different Branches of the PV

Flow resistance analyses were further conducted for different branches of the PV system to investigate which part is sensitive to cirrhosis patients with CSPH. In this section, the flow resistance of the SV, SMV, MPV, LPV, and RPV will be discussed.

**Figure 4:**
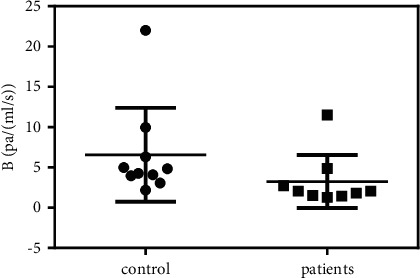
Flow resistance analysis of SV.

A comparison of the flow resistance coefficient *B* in SV is presented in Figure 4. As shown, the *B* of the patients (3.2 ± 1.0 Pa/(mL/s)) was much lower than that of the control group (6.5 ± 1.8 Pa/(mL/s)). Further *t*-test showed *P*=0.07, close to the statistical significance between the two groups. The SV transports blood from the spleen and pancreas to the liver. When cirrhosis occurs, the high flow resistance inside the liver obstructs bloodstreams from entering the liver and results in the SV dilation and flow resistance reduction.

**Figure 5:**
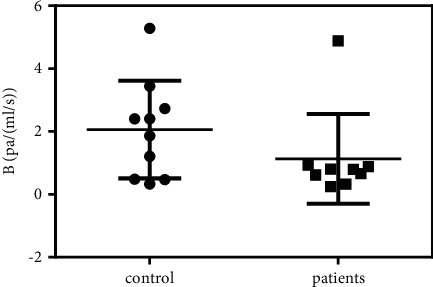
Flow resistance analysis of SMV.

A comparison of the flow resistance coefficient *B* in SMV is presented in Figure 5. As shown, the *B* of the patients (1.13 ± 0.48 Pa/(mL/s)) was lower than that of the control group (2.66 ± 0.50 Pa/(mL/s)). Nevertheless, the results of the *t*-test showed *P*=0.09, proving that the difference between the two groups was close to statistically significant.

**Figure 6:**
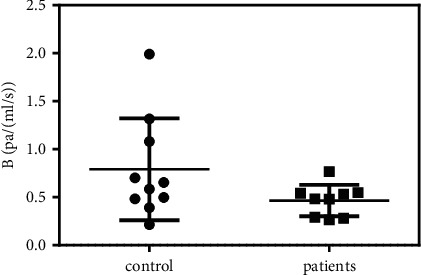
Flow resistance analysis of MPV.

A comparison of the flow resistance coefficient *B* in MPV is presented in Figure 6. The flow resistance coefficient *B* of the patients (0.46 ± 0.05 Pa/(mL/s)) was lower than that of the control group (0.79 ± 0.17 Pa/(mL/s)), and this difference was statistically significant (*P*=0.04).

**Figure 7:**
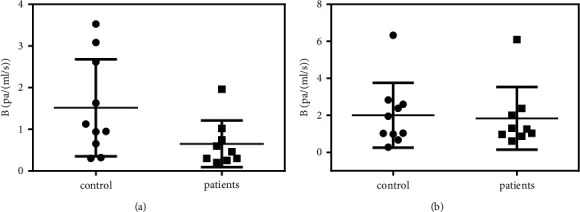
Flow resistance of LPV and RPV: (a) LPV (b) RPV.

The flow resistance coefficients *B* of the LPV are presented in Figure 7(a). As shown, the *B* (0.65 ± 0.19 Pa/(mL/s)) of the patients was much lower than that of the control group (1.51 ± 0.37 Pa/(mL/s)). Further *t*-tests showed *P*=0.02, indicating statistical significance. The flow resistance coefficients B of the RPV are presented in Figure 7(b). As shown, the *B* (1.83 ± 0.56 Pa/(mL/s)) of the patients was lower than that of the control group (2.00 ± 0.55 Pa/(mL/s)). Further *t*-tests showed *P*=0.41, confirming no statistical significance between the two groups. The results above confirmed that in the patient group the dilations in the LPV were more advanced than those in the RPV; this may result in uneven distributions of blood flow in the left and right lobes that matched those observed in earlier studies [26].

Flow resistance coefficients B at different branches are summarized in Table 2. What stands out in this table is the flow resistance reduction occurred in every branch of the PV system in patients. Among them, the MPV and LPV flow resistance reductions were statistically significant.

## 4. Discussion

As mentioned in the introduction section, image-based noninvasive diagnostic methods are urgently needed for detecting CSPH in cirrhosis patients. A CFD-based flow resistance analysis that uses CFD to measure pressure drops at different volumetric flow rates and summarizes the constant flow resistance coefficients for a certain vessel is proposed in this paper. The flow resistance coefficient reflects the overall effect of the vessel morphology on the blood flow and hence is sensitive to all types of patients, including patients with minor vessel dilation.

Based on the enhanced CTA images, the CFD method was used to estimate the flow resistances at the whole PV system and each branch. The results showed that the flow resistance of the whole PV system was reduced in cirrhosis patients with CSPH; among all branches, the MPV and LPV contributed the most to the flow resistance reduction.

Due to the increase of hepatic pressure, the vessel dilation of the PV system is often found in cirrhosis patients with CSPH [27], and the cross-sectional area of MPV is a commonly used indicator for detecting cirrhosis patients with CSPH. Nevertheless, the cross-sectional area analysis is operator-dependent and only measures the vessel locally, therefore, unlikely to identify CSPH patients with no obvious MPV dilation. In this study, nine cirrhosis patients with CSPH and ten participants without liver diseases were examined using the flow resistance analysis and the cross-sectional area measurement. The results showed that compared to the cross-sectional area analysis, the proposed CFD-based flow resistance analysis exhibits high repeatability and high sensitivity in the detection of CSPH. Thereby, we suggest using flow resistance analysis as a supplementary method to detect CSPH in clinical practice.

We should emphasize that the sample size of the patients was relatively small and we still used Baveno VI but not the updated Baveno VII [28] for the determination of CSPH patients. Those are the main limitations of this study. In future work, the proposed flow resistance-based method needs to be further verified with more cases and with more strict criteria of CSPH.

## 5. Conclusions

- Cirrhosis-induced CSPH causes portal vein dilation and results in the flow resistance decrease in the PV system and its branches.
- Flow resistance coefficient B of the PV system can be potentially used to identify cirrhosis patients with CSPH.
- Analysis of different branches showed that the statistically significant decrease of flow resistance in cirrhosis patients with CSPH first happened in the MPV and LPV branches.

## Figures and Tables

**Table 1:** Main characteristics of the patients.

	All patients
Gender (male-female) %	7 (77.8%)-2 (22.2%)

Age (years)	63.5 [42-91]

Cause of cirrhosis	
Alcohol	2 (22.2%)
Viral hepatitis	6 (66.7%)
Cholestatic disease	1 (11.1%)

BMI (kg/m^2^)	23.9 [19.8-29.1]
Liver stiffness (kPa)	25.5 [20.4-43.4]
Platelets (10 ^ 9/L)	125 [39.0-388.0]
Prothrombin time (%)	56 [29.4-97.6]
Serum albumin (g/L)	34.6 [27.2-46.1]
AST (IU/L)	125.1 [31.7-638.4]
ALT (IU/L)	146.5 [14.0-908.6]

Collateral circulation	
Yes	5 (55.6%)
No	4 (44.4%)

**Table 2:** Flow resistance coefficients (*B*) at different branches.

	Coefficient *B* Pa/(mL/s)		
	Cirrhosis group	Control group	*P*
SV	3.20 ± 1.00	6.50 ± 1.80	0.07
SMV	1.13 ± 0.48	2.66 ± 0.50	0.09
MPV	0.46 ± 0.05	0.79 ± 0.17	0.04
LPV	0.65 ± 0.19	1.51 ± 0.37	0.02
RPV	1.83 ± 0.56	2.00 ± 0.55	0.41

## Data Availability

The data used to support the findings of this study are not available due to ethical restrictions.
